# Diet and temperature modify the relationship between energy use and ATP production to influence behavior in zebrafish (*Danio rerio*)

**DOI:** 10.1002/ece3.7806

**Published:** 2021-06-21

**Authors:** Amélie Le Roy, Geoffrey P. F. Mazué, Neil B. Metcalfe, Frank Seebacher

**Affiliations:** ^1^ School of Life and Environmental Sciences University of Sydney Sydney NSW Australia; ^2^ Institute of Biodiversity, Animal Health and Comparative Medicine University of Glasgow Glasgow UK

**Keywords:** boldness, exploration, food availability, mitochondria, oxygen consumption, P:O ratio

## Abstract

Food availability and temperature influence energetics of animals and can alter behavioral responses such as foraging and spontaneous activity. Food availability, however, is not necessarily a good indicator of energy (ATP) available for cellular processes. The efficiency of energy transduction from food‐derived substrate to ATP in mitochondria can change with environmental context. Our aim was to determine whether the interaction between food availability and temperature affects mitochondrial efficiency and behavior in zebrafish (*Danio rerio*). We conducted a fully factorial experiment to test the effects of feeding frequency, acclimation temperature (three weeks to 18 or 28°C), and acute test temperature (18 and 28°C) on whole‐animal oxygen consumption, mitochondrial bioenergetics and efficiency (ADP consumed per oxygen atom; P:O ratio), and behavior (boldness and exploration). We show that infrequently fed (once per day on four days per week) zebrafish have greater mitochondrial efficiency than frequently fed (three times per day on five days per week) animals, particularly when warm‐acclimated. The interaction between temperature and feeding frequency influenced exploration of a novel environment, but not boldness. Both resting rate of producing ATP and scope for increasing it were positively correlated with time spent exploring and distance moved in standardized trials. In contrast, behavior was not associated with whole‐animal aerobic (oxygen consumption) scope, but exploration was positively correlated with resting oxygen consumption rates. We highlight the importance of variation in both metabolic (oxygen consumption) rate and efficiency of producing ATP in determining animal performance and behavior. Oxygen consumption represents energy use, and P:O ratio is a variable that determines how much of that energy is allocated to ATP production. Our results emphasize the need to integrate whole‐animal responses with subcellular traits to evaluate the impact of environmental conditions on behavior and movement.

## Graphical abstract

Food availability and temperature influence energetics of animals and can alter behavioral responses. We show that infrequently fed zebrafish have greater mitochondrial efficiency than frequently fed animals, particularly when warm‐acclimated. The interaction between temperature and feeding frequency influenced exploration of a novel environment, but not boldness.

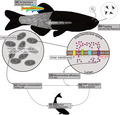

## 1 INTRODUCTION

Animal behavior is linked to energetics and metabolism and thereby also to temperature changes (Abram et al., 2017). Environmental variation modulates the interaction between physiology and behavior, and variation in temperature or food availability can potentially exacerbate their influence on behavioral traits such as activity and boldness (Killen et al., 2013). Availability of food resources, in particular, can modify behavioral choices in a manner that is dependent on the energetic status of the animal (Dall & Johnstone, 2002). The motivation for animals to move is often linked to their energy status, and food availability and intake influence behavior such as foraging, risk taking, and exploration of novel environments (Krause et al., 1998). For example, hungry animals tend to move more often and greater distances in search of food, and take greater risks (Killen et al., 2011). The quantity of available food and its predictability in a particular habitat will therefore also modify the spatial ecology of animals (Riotte‐Lambert & Matthiopoulos, 2020). However, food availability does not necessarily reflect energy (ATP) availability at the cellular level. There are numerous processes involved in transforming ingested food to chemical energy, such as digestion and assimilation (Barneche & Allen, 2018), which influence energy conversion efficiency. However, the electron transport chain in mitochondria is the most proximal and one of the most important sites that influences energy transduction efficiency (Brand, 2005).

Food is converted into substrates (NADH and FADH_2_) that are used by mitochondria to produce ATP. Electrons from substrates are passed between protein complexes in the inner mitochondrial membrane, thereby releasing energy that is used to pump protons from the mitochondrial matrix across the inner membrane. Oxygen is used as the final electron acceptor in this electron transport chain. The energy stored in the proton gradient powers the conversion of ADP into ATP by the ATP synthase (complex V) (Jastroch et al., 2010). However, not all energy contained in the proton gradient is used to make ATP. A substantial proportion of energy (up to 30%) can be lost by protons returning to the mitochondrial matrix outside the ATP synthase (Brand, 2005; Jastroch et al., 2010). Hence, food‐derived substrate oxidation and oxygen consumption rates do not necessarily reflect ATP production rates. Mitochondrial efficiency (i.e., ADP used or ATP produced for each oxygen consumed =P:O ratio) can vary between individuals or with environmental differences such as in temperature or food availability (Salin et al., 2015; Trzcionka et al., 2009). Increased mitochondrial efficiency comes at the cost of excess reactive oxygen species production, which can damage membranes, proteins, and DNA (Salin et al., 2015). Hence, mitochondrial efficiency is not always maximized, but it is an essential component in determining energy‐consuming processes at the whole‐animal level.

Decreasing temperature has a negative thermodynamic effect on reaction rates. However, thermal acclimation can shift the acute thermal sensitivity of physiological rates and can thereby buffer physiological rates from environmental variation (Schulte et al., 2011). For example, cold‐acclimated fish often have increased rates of mitochondrial substrate oxidation (State 3 rates) which may promote efficiency, although the increase in substrate oxidation can be accompanied by increased rates of proton leak (State 4 rate) (Khan et al., 2014). Prolonged (> weeks) exposure to different thermal conditions can thereby modify the relationship between ATP production and oxygen consumption at different acute temperatures. However, the direction in which temperature influences mitochondrial energetics is not resolved. For example, acute increases in temperature reduced efficiency of toad (*Bufo bufo*) mitochondria (Roussel & Voituron, 2020), but warm acclimation increased mitochondrial efficiency in mosquitofish (*Gambusia holbrooki*) (Le Roy & Seebacher, 2020). In contrast, warm acclimation decreased mitochondrial efficiency in polar cod (*Boreogadus saida*), but did not affect it in Atlantic cod (*Gadus moruha*) (Leo et al., 2017).

In addition to temperature, food availability can modify mitochondrial bioenergetics. For example, brown trout (*Salmo trutta*) mitochondria showed increased maximal substrate oxidation (state 3) rates and decreased proton leak (state 4) rates in response to fasting, thereby increasing ATP production efficiency (Salin et al., 2018). Similarly, P:O ratios of skeletal muscle mitochondria were on average 15% higher in fasting penguin chicks compared to fed chicks, which presumably compensated for scarcity of resources (Monternier et al., 2014). Mitochondrial efficiency may thereby influence behavior, because greater efficiency means that reduced food availability or increased ATP use do not necessarily lead to negative energy status.

**FIGURE 1:**
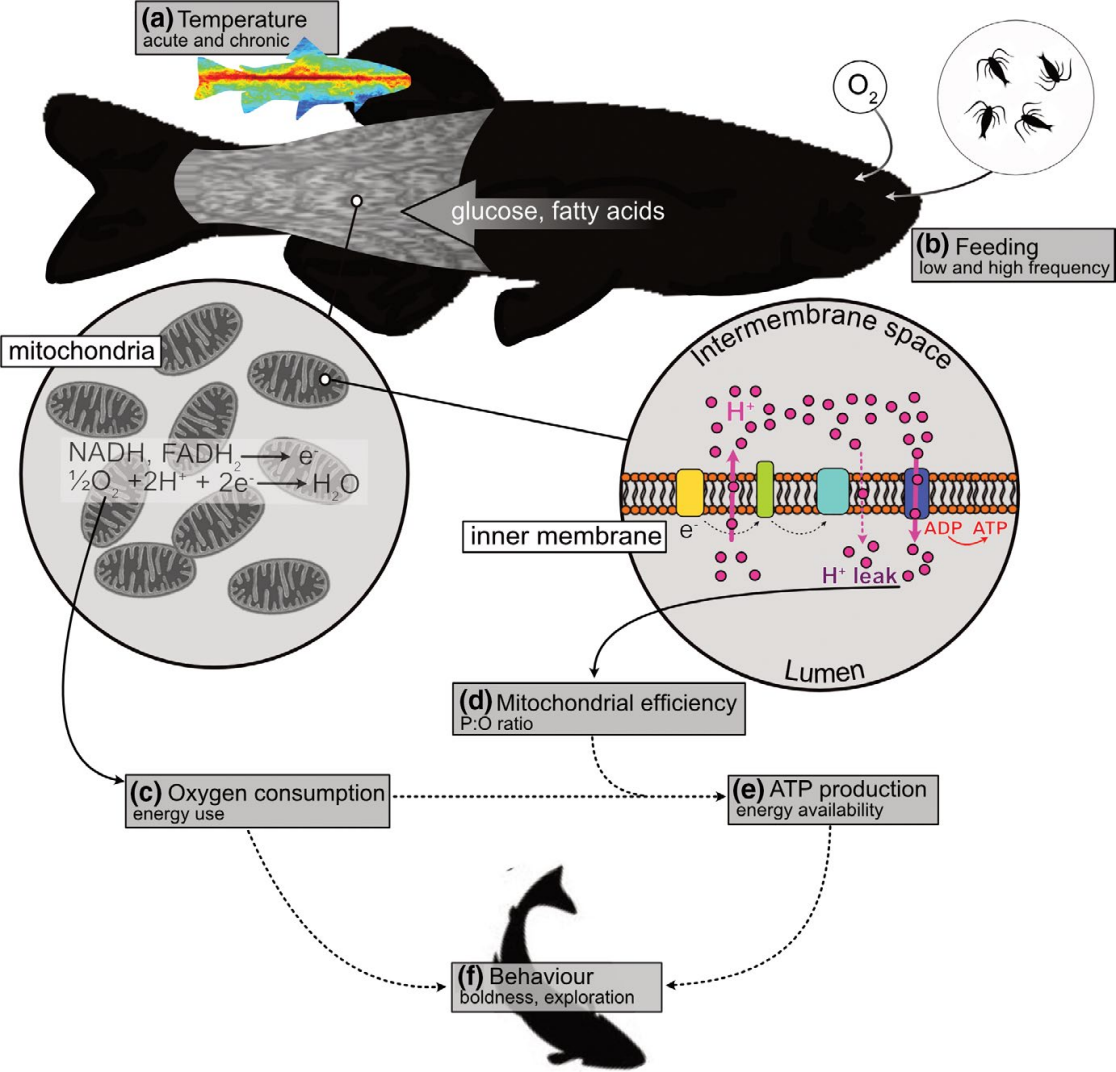
Conceptual outline of the study. Food availability does not necessarily indicate energy available for cellular work, at least partly because of variation in mitochondrial efficiency. We tested whether different acute and acclimation temperatures (a) interact with food availability (b) to alter energy transduction efficiency and thereby influence behavior. Food is broken down into macronutrients, and glucose and fatty acids are principally responsible to yield substrates (NADH and FADH_2_) oxidized in muscle mitochondria. Whole‐animal oxygen consumption (c) is therefore a measure of the energy used in the mitochondrial electron transport chain. However, the efficiency of mitochondria (d) in converting the energy released by substrate oxidation into ATP (P:O ratio) can vary with environmental conditions. To estimate the amount of ATP produced, we corrected oxygen consumption for mitochondrial efficiency (e). This procedure permitted us to test the relative importance of increased energy use or increased ATP availability in influencing behavior (f), for example, by stimulating or reducing foraging, respectively. Note that the broken arrows indicate connections between experimental measures rather than biological cause‐and‐effect relationships

Our aim was to determine whether the interaction between food availability and temperature affects mitochondrial efficiency and behavior in zebrafish (*Danio rerio*). We conducted a fully factorial experiment to test for interactions between food availability (infrequent or frequent feeding), acclimation temperature (three weeks at 18 or 28°C), and acute test temperature (18 or 28°C). We measured whole‐animal oxygen consumption, mitochondrial bioenergetics, and behavior (boldness and exploration) in individual zebrafish (*Danio rerio*; Figure 1). We hypothesized that (i) infrequently fed fish have higher P:O ratios, but nonetheless show greater foraging motivation indicated by greater boldness and exploratory behavior than frequently fed fish; (ii) oxygen consumption is often interpreted as representing ATP production, but cold acclimation decreases P:O ratios, so that oxygen consumption overestimates ATP production, and there is an interaction between acclimation and feeding frequency; note that the directionality of this hypothesis could be reversed because there is no consensus in the literature. (iii) Warm‐acclimated fish consume more energy and therefore show greater boldness and exploratory behavior than cold‐acclimated fish, although this relationship may be offset by decreased P:O ratios in cold‐acclimated fish. A corollary from (iii) is that (iv) resting oxygen consumption is positively correlated with boldness and exploration because higher energy consumption requires greater foraging particularly when food is limited (Krause et al., 1998). Conversely, ATP production should be negatively related to these behavioral traits because greater efficiency of mitochondria would mean that fish have to forage less. (v) Greater energy use during movement could lead to reduced exploration, particularly when food is limited. We therefore predict that more efficient ATP supply is associated with exploration.

## 2 MATERIALS AND METHODS

### 2.1 Study design

All experiments were carried out with the approval of the University of Sydney Animal Ethics Committee (approval number: 2017/1200). Adult short‐fin zebrafish (*Danio rerio*; mean standard length = 3.27 ± 0.026 [*SE*] cm; mean mass = 0.48 ± 0.014 [*SE*] g) were obtained from a commercial supplier (Livefish, Bundaberg, Australia) and maintained in plastic tanks (600 × 450 × 250 mm; 1–2 fish/l) at 23°C and with a photoperiod of 14 hr light:10 hr dark; these temperature and light conditions corresponded to holding conditions at the supplier. Each tank contained a sponge filter (Age of Aquariums, Australia) connected to an air pump (AC‐9908, Resun, China), and submersible heaters (200W, AquaWorld, Australia) maintained the water temperature within 0.5°C of the desired temperature. After one week, fish were split randomly across two temperature treatments (18°C or 28°C) and acclimated to these conditions for three weeks. We chose these acclimation temperatures because they fall within the natural range experienced by the species, but do not represent extremes (Spence et al., 2007). During the temperature acclimation, half of the fish from each temperature treatment were fed fish flakes (Supervit Tropical Fish Flakes, Tadeusz Ogrodnik, Chorsów, Poland) once per day four times a week (diet treatment: infrequently fed fish). The other half was fed three times per day on five days a week (diet treatment: frequently fed fish). Fish were fed 5 mg of flakes per fish at each feeding time, and the days on which fish were fed was randomized in both treatments so that the infrequently fed fish received lower amounts of food and food supply was less predictable. Hence, there were four treatments (*N* = 16–17 fish per treatment), and we dispersed fish across five experimental tanks (34 × 19 × 25 cm; 4–6 fish per tank) per treatment. Fish were weighed, and standard length was measured at the beginning of the treatments and at the end of treatments when fish were euthanized for mitochondrial measurements. We calculated a refinement of Fulton's *k* condition factor (Nash et al., 2006), regressing log(mass) against log(length) to obtain the exponent (2.55) of the linear dimension, so that *k* = mass/length^2.55^. Our aim was to maintain fish within a healthy weight range (*k* = >1.8 and <3) but with a negative (infrequently fed) and positive (frequently fed) energy balance. Hence, we aimed to avoid starvation (*k* < 1.8) (McClelland et al., 2006) or obesity (*k* > 3) (Seebacher et al., 2017). The feeding regime was based on our experience in working with obese zebrafish (Seebacher et al., 2017). Fish in the infrequently fed group lost some weight and the frequently fed fish gained weight during the treatments, but all fish remained within an acceptable range (Table S1).

After three weeks of treatments, we measured behavior, resting and maximum oxygen consumption rates, and mitochondrial bioenergetics at two acute test temperatures (18°C and 28°C); all responses were measured in each individual fish, and fish used for measurements were chosen randomly from the treatment tanks. There were at least 24 hr between behavioral and maximum metabolic rate measurements at different test temperatures within each fish, and the order of acute test temperatures was switched between fish to avoid order effects in all trials. All measurements were made within 4 days for each fish. We tracked individuals between the different measurements by keeping individual fish within 1 L perforated cylindrical baskets within their home tanks. The baskets permitted water flow and hence visual and olfactory contact between fish, but prevented egress of the fish (Loughland & Seebacher, 2020). To ensure that fish remained in their respective treatments for similar periods of time, we conducted three blocks of treatments over five weeks, each time implementing all treatments but with reduced sample sizes.

### 2.2 Behavioral trials

We measured latency to leave a refuge (boldness) and exploration of a novel environment in four identical experimental arenas. Arenas were made of opaque white Perspex (118 cm L × 30 cm W × 20 cm H, with 7 cm water depth) and divided into two distinct compartments by an opaque plastic partition. The refuge compartment (20 × 30 × 20 cm) was covered by an opaque plastic sheet to provide shelter for the fish. The adjoining, uncovered novel “exploration” compartment (98 × 30 × 20 cm) was enriched with six submerged, colored structures (blue, red, and green) with a square base (8 × 8 cm) and sloping edges to a height of 2.5 cm. The distribution pattern of the colored structures was changed between repeated trials at different test temperatures so that fish experienced a novel environment at each trial (Mazue et al., 2015). The two compartments were separated by a removable gate (8 × 10 cm) made of white plastic corflute. At the start of each behavioral assay, fish were introduced into the refuge compartment and left undisturbed for 10 min. We then opened the gate remotely without disturbing the fish. We filmed the arena (using a G1X, Canon camera filming at 30 fps at a resolution of 1,080 × 720 pixels), and from the video, we determined the latency to emerge from the refuge and enter the exploratory compartment for the first time (“time to leave refuge”) as a measure of boldness (Krause et al., 1998). Individuals that did not leave the refuge within 20 min were given a maximum latency time of 1,200 s and were not scored for exploration (a total of seven fish did not leave the refuge: three at 18°C and four at 28°C, four from the frequently fed treatment, and three from the infrequently fed treatment). We selected the 10 min (18,000 frames) of the video recording immediately after the fish left the refuge to quantify exploration tendency. Cropped video files were converted to.avi (using VIRTUALDUB version 1.10.4) before being uploaded to the CTRAX automated tracking software (Caltech Ethonomics Project, The Caltech Multiple Fly Tracker, Version 0.5.18) (Branson et al., 2009). The x, y coordinates of each individual were hand‐corrected using the FIXERRORS GUI in MATLAB (Branson et al., 2009) so that each excursion to the exploration arena was recorded as a single unbroken time series. We extracted the total distance traveled in the exploration arena (“distance”), and the total time spent in the exploration arena (“time spent exploring”) for analysis.

### 2.3 Whole‐animal oxygen consumption

Resting oxygen consumption was measured by closed system respirometry according to published protocols (Le Roy et al., 2017). Fish were not fed for 24 hr before measures of oxygen consumption. Fish were placed in Perspex cylindrical respirometers (15 mm diameter × 100 mm length, 27 ml volume) and immersed in a temperature‐controlled water bath. Respirometers were connected to a peristaltic pump (i150, iPumps, Tewkesbury, UK), which circulated water through the chambers. Oxygen concentration inside the chambers was measured using sensor spots (Loligo Systems, Viborg, Denmark) stuck to the inside, halfway along the length of the chambers and monitored by fiber optic cables linked to an oxygen meter (Witrox, Loligo Systems, Viborg, Denmark). We monitored 15 fish in individual chambers concurrently. Fish were allowed to rest for two hours before measurements, which is sufficient time to overcome handling stress (Seebacher et al., 2016), before pumps were turned off and the decrease in oxygen concentration inside the sealed chamber was monitored for 30 min; oxygen content did not fall below 80% saturation during any trial. After the 30 min recording period, we turned the peristaltic pump on again and gradually over 30 min changed the water temperature to the second test temperature. After the fish spent 15 min at the new test temperature, we measured oxygen consumption as above. During the trials, fish were monitored with a camera connected to the data acquisition computer to ensure that resting levels of oxygen consumption were recorded in stationary fish.

After measuring resting oxygen consumption and on the same day, we determined exercise‐induced (maximal) rates of oxygen consumption in glass respirometers (130 ml) containing a magnetic stir bar that was separated from the fish by a mesh partition. A central plastic column suspended from the lid inside the chamber helped reduce turbulence. The chamber was immersed in a temperature‐controlled water bath, which was placed on top of a magnetic stirrer. We controlled the circular flow in the chamber by adjusting the speed on the magnetic stirrer. A sensor spot (Loligo Systems, Viborg, Denmark) glued to the inside of the chamber wall measured oxygen concentration and was monitored by a fiber optic cable, connected to an oxygen meter (FIBOX 3, PreSens, Regensburg, Germany). We increased the flow speed inside the chamber gradually over 30 s until the fish struggled to hold its position in the water column, which we considered to be near maximal swimming speed. We recorded the decrease in oxygen concentration at this flow speed for approximately 10 min, and oxygen concentrations always remained >90% saturation during the trials. We extracted the slopes from the resting and maximal oxygen concentration curves to calculate oxygen consumption rates (in µmol O_2_ g^−1^ min^−1^) in each individual. We calculated aerobic scope as the difference between maximal and resting rates of oxygen consumption. At the end of the measurements of whole‐animal oxygen consumption, the fish were returned to their treatment tanks.

### 2.4 Mitochondrial bioenergetics

Within a week of measuring whole‐animal oxygen consumption (during which fish were kept in their treatments), fish were euthanized by cervical dislocation after anesthesia in a buffered MS222 solution (0.4 g/l; Sigma‐Aldrich, Castle Hill, NSW, Australia). After measuring and weighing fish, skeletal muscle was dissected on ice for measurements of mitochondrial respiration. Mitochondrial measurements were conducted at 18 and 28°C consecutively for each sample. All chemicals were purchased from Sigma‐Aldrich (Castle Hill, Australia). The dissected tail muscle was homogenized using a Potter–Elvehjem tissue homogenizer in nine volumes of isolation buffer (KCl 140 mM, HEPES 20 mM, MgCl_2_ 5 mM, EGTA 2 mM, ATP 1 mM, BSA [fatty acid free] 0.5 g/l, pH 7) (DosSantos et al., 2013). The homogenate was centrifuged at 1,400 *g* for 5 min, and the supernatant containing the mitochondria was set aside while the pellet was resuspended and centrifuged again. The supernatants were combined and centrifuged at 9,000 *g* for 9 min to settle the mitochondria, and the supernatant was discarded. The pellet was resuspended in assay medium (sucrose 110 mM, KCl 60 mM, EGTA 0.5 mM, MgCL_2_ 3 mM, taurine 20 mM, KH_2_PO_4_ 10 mM, HEPES 20 mM, BSA‐FAF 0.5 g/l, pH7.1) (DosSantos et al., 2013) at a ratio of 2 ml/g of tissue. Mitochondrial oxygen consumption was measured in a respiration chamber (Mitocell MT200; Strathkelvin Instruments, North Lanarkshire, UK) with an oxygen electrode (model 1302; Strathkelvin Instruments) connected to an oxygen meter (model 782; Strathkelvin Instruments). We triggered state 2 respiration by adding a final concentrations of 5 mM malate and 2.5 mM pyruvate. State 3 respiration was induced by adding 0.1 mM ADP, and we determined natural state 4 when ADP was used up, indicated by stabilization of oxygen consumption rate 30–50 min after ADP addition. Finally, the addition of 1 µM of carbonyl cyanide‐p‐trifluoromethoxy phenylhydrazone (FCCP) estimated the maximal uncoupled rates of oxygen uptake and allowed us to verify the integrity of the mitochondrial membrane, that is, FCCP “punctures” the membrane so that oxygen consumption should increase after its addition to mitochondria with intact membranes. Protein concentration of the mitochondrial extract was determined using a Bradford assay (Sigma‐Aldrich, Castle Hill, Australia), with BSA as a standard. Mitochondrial efficiency was assessed as the P:O ratio, a proxy of which was calculated as the amount of ADP added to the chamber (250 or 125 μM final concentration) divided by the number of oxygen atoms used to consume it; while this does not allow instantaneous calculation of the P:O ratio, it is a relative measure that allows comparisons between treatments. Oxygen use was estimated as the difference in oxygen levels between the beginning of state 3 and the start of state 4. Whole‐animal oxygen consumption reflects activity of mitochondrial complex IV (cytochrome c oxidase), which uses oxygen as the final electron acceptor. Oxygen consumption is therefore proportional to food‐derived substrate (NADH and FADH_2_) oxidation in the electron transfer chain. However, not all energy released by substrate oxidation (and stored in the mitochondrial proton gradient) is converted into ATP by complex V (ATP synthase), because of proton leak, and varying amounts of protons pumped for each pair of electrons transferred, for example, (Brand, 2005). P:O ratios are an intrinsic characteristic of mitochondria indicating how many ADP molecules are converted to ATP per oxygen atom used. P:O ratios therefore can be used to estimate the proportion of substrate oxidized (indicated by oxygen consumed) that is used for ATP production (Salin et al., 2015). Hence, we generated an estimate of ATP production by multiplying oxygen consumption of individual fish by their corresponding P:O ratio. From our hypotheses, we specifically analyze ATP production at rest (i.e., resting oxygen consumption × P:O ratio; ATP_rest_) and ATP production scope (i.e., aerobic [oxygen consumption] scope × P:O ratio; ATP_scope_). Note that in the text we refer to the difference between maximal and resting rates of whole‐animal oxygen consumption as “aerobic scope,” which is distinct from “ATP production scope.” In our approach, we assume that there is no ADP in the mitochondrial preparation other than what we added experimentally and that most ADP is converted to ATP. However, P:O ratios may be overestimated if not all ADP is used up, but any resulting variation should not modify treatment effects. We measured muscle mitochondria only, and there may be differences in mitochondrial characteristics between tissues. However, myotomal muscle makes up >60% of the body mass in fish (Johnston et al., 2011) so that skeletal muscle metabolism is relevant for whole‐animal energetics.

### 2.5 Statistical analysis

We analyzed all data with permutational analyses using the R package “lmPerm” (Wheeler & Torchiano, 2016), because it uses the data per se for analysis and is free from assumptions about underlying distributions (Drummond & Vowler, 2012). We used diet (infrequently and frequently fed), acclimation temperature (18°C and 28°C), and test temperature (18°C and 28°C) as fixed factors to determine their effect on mitochondrial bioenergetics and efficiency, whole‐animal oxygen consumption, and behavior. The identity of individual fish was used as a random factor to account for the repeated measures across different test temperatures. We used mass as a covariate for oxygen consumption rates, and it was not significant in any case indicating lack of allometry across the size range of the fish we used. In the analysis of behavioral responses, we used condition factor as a covariate to control for potential effects of differences in body shape per se which may affect locomotor behavior. A reviewer suggested to analyze boldness and exploration with a principal component analysis, but we prefer to report these responses separately because they are standard measures in the literature.

We tested the specific hypotheses (see Introduction) about the relationships between metabolic characteristics and behavior by including resting oxygen consumption (MO_2rest_), aerobic scope (MO_2scope_), ATP production at rest (ATP_rest_), and scope for ATP production (ATP_scope_) as covariates in a factorial permutational analyses. Because we proposed specific hypotheses about the effect of frequency of feeding on these relationships, we conducted separate analyses for frequently and infrequently fed fish to determine the significance of the covariates. Threshold for significance was α < 0.05.

Note that we chose to present mean resting and maximal rates of oxygen consumption, and mean state 3 and state 4 rates of mitochondrial respiration in the same Figure panels, respectively, to facilitate comparisons between these rates, rather than plotting marginal means in separate panels as suggested by a reviewer. We report eta‐squared values (*η* ^2^) as estimators of effect sizes as requested by a reviewer.

## 3 RESULTS

### 3.1 Resting and maximum oxygen consumption rates increased with cold acclimation and with frequency of feeding

**FIGURE 2:**
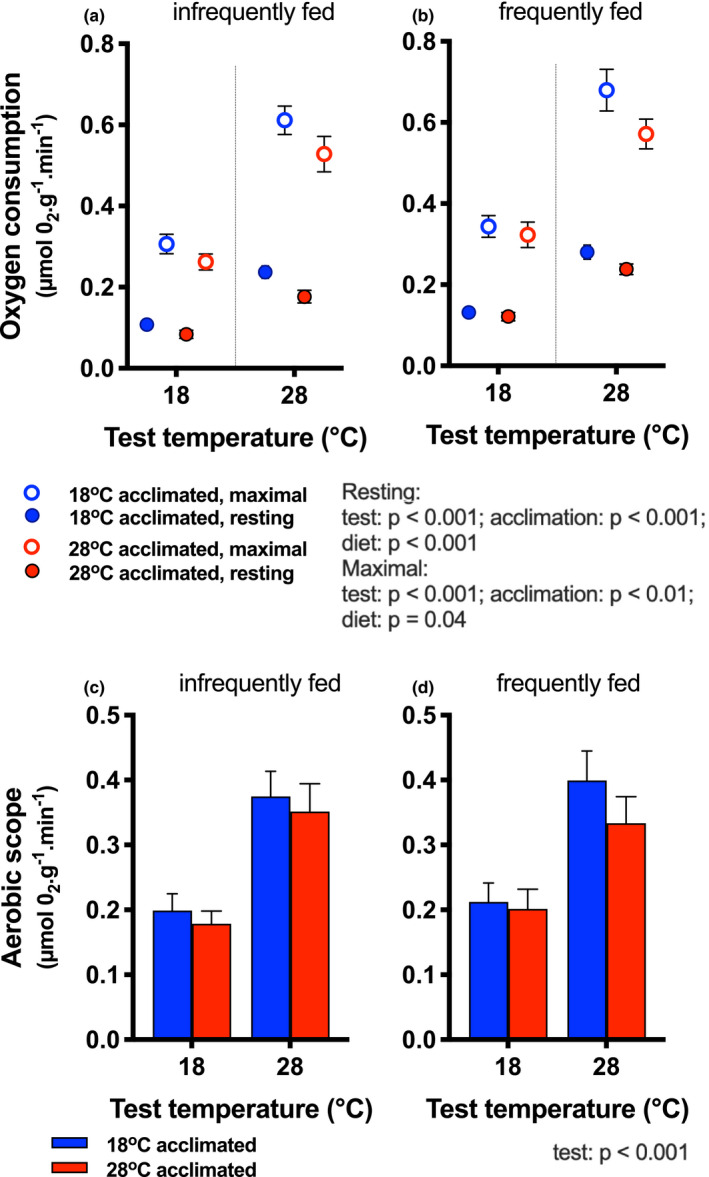
Whole‐animal oxygen consumption. Resting (filled circles) and maximal (open circles) oxygen consumption rates are shown for fish infrequently (a) and frequently fed (b). Test temperature, acclimation temperature, and diet each had main effects on resting and maximal rates (blue symbols = 18°C acclimation; red symbols = 28°C acclimation) for resting rates. Aerobic scope [(c) infrequently fed, (d) frequently fed] was higher at 28°C test temperature than at 18°C, and none of the other factors (blue bars = cold‐acclimated; red bars = warm‐acclimated) had a significant effect. Statistical results are summarized next to the legend. Means ± *SE* are shown and sample size was *n* = 16–17 fish per treatment group. Thin vertical lines in (a) and (b) are to help visual clarity only

Both resting and maximal rates of oxygen consumption were higher in frequently fed fish (main effect of diet, resting rates *η* = 0.061; maximal rates *η* ^2^ = 0.016; Table 1; Figure 2a,b), in cold‐acclimated fish (main effects of acclimation; resting rates *η* ^2^ = 0.041; maximal rates *η* ^2^ = 0.024), and at the higher test temperature (main effect of test temperature; resting rates *η* ^2^ = 0.5 maximal rates *η* ^2^ = 0.47; Table 1; Figure 2a,b).

**TABLE 1:** Results from the analysis of whole‐animal oxygen consumption and mitochondrial bioenergetics. Results from the permutational analysis are shown for state 3 mitochondrial respiration rate (S3), state 4 rate (S4), P:O ratios (P:O), resting oxygen consumption rate (Rest), maximal oxygen consumption rate (Max), aerobic scope (MO_2scope_), ATP production at rest (ATP_rest_), and net ATP production (maximum rest; ATP_scope_). Numerator degrees of freedom = 1 for all factors and interactions (Test = acute test temperature; Acc = acclimation temperature; Diet = frequency of feeding), and sample sizes were 16–17 fish for each treatment group. Significant results are shown in bold, and *df* = 1, 62 in all cases; see main text for more details of analysis

Source	S3	S4	P:O	Rest	Max	MO_2scope_	ATP_rest_	ATP_scope_
TestT	**<0.01**	**0.02**	0.36	**<0.001**	**<0.001**	**<0.001**	**<0.001**	**<0.001**
AccT	0.21	0.25	**<0.001**	**<0.001**	**<0.01**	0.32	0.40	**0.01**
Diet	0.17	0.37	**<0.001**	**<0.001**	**0.04**	0.41	0.31	**<0.001**
TestT:AccT	0.88	0.38	0.42	0.079	0.20	0.84	0.98	0.38
TestT:Diet	0.58	0.16	0.92	0.44	0.98	1	0.92	1
AccT:Diet	0.64	0.21	**<0.001**	0.68	1	0.69	0.52	**<0.001**
TestT:AccT:Diet	0.62	0.43	0.32	0.80	0.58	0.78	1	0.39

### 3.2 Low food availability and warm acclimation increased mitochondrial efficiency

**FIGURE 3:**
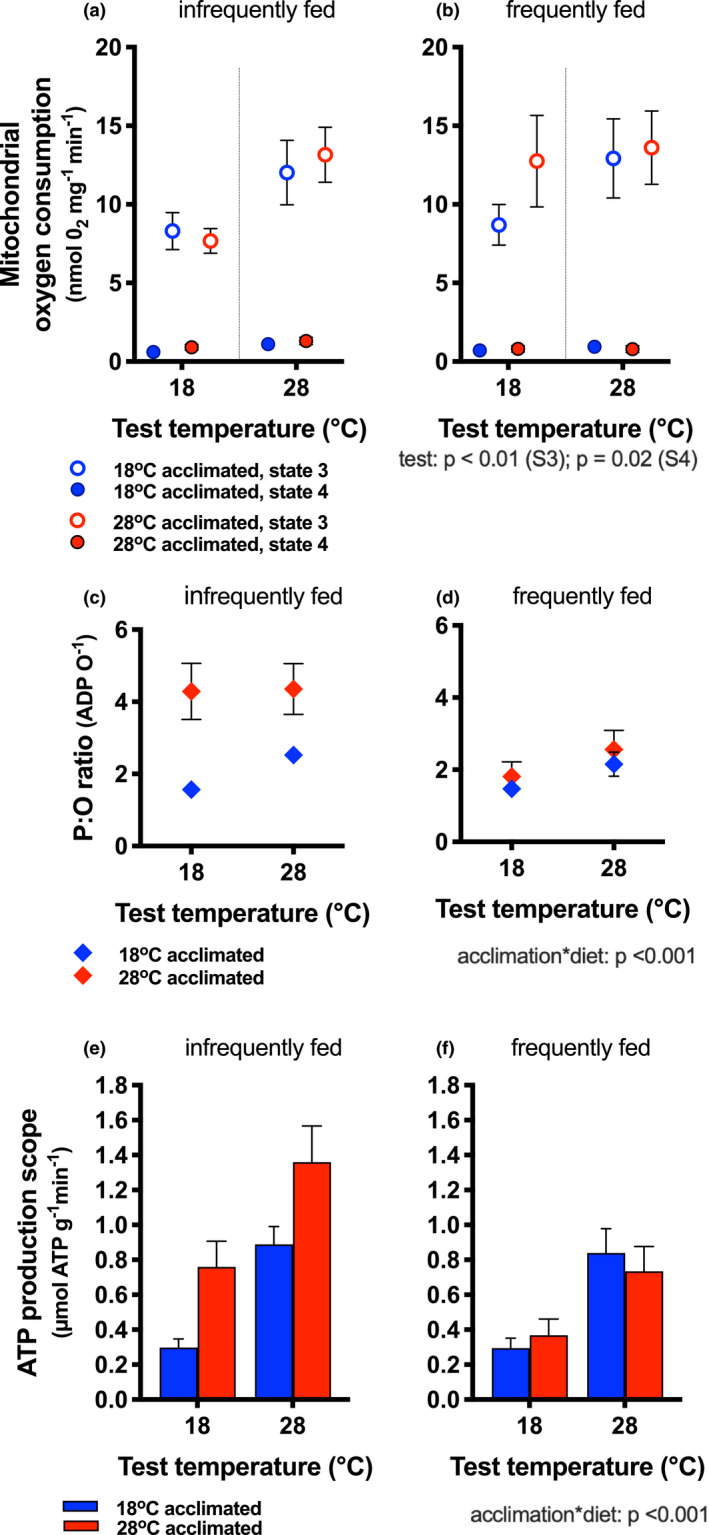
Mitochondrial bioenergetics and scope for ATP production. State 3 (open circles) and state 4 (filled circles) rates of mitochondrial oxygen consumption in fish fed infrequently (a) and frequently (b) and acclimated to 18°C (blue symbols) and 28°C (red symbols). Rates of oxygen consumption increased with increasing test temperature (main effect, indicated next to legend), but diet and acclimation temperature had no effect on state 3 or state 4 rates. P:O ratios (ATP produced for each oxygen consumed) increased with acclimation temperature and diet, but was highest in 28°C acclimated animals (red bars) that were fed infrequently compared to cold‐acclimated fish (blue bars) and those fed frequently (diet*acclimation interaction; indicated next to legend). ATP production scope increased with increasing test temperature [(e) infrequently fed, (f) frequently fed], and it increased particularly in warm‐acclimated fish that were infrequently fed (acclimation* diet interaction). Means ±* SE* are shown, and sample size was *n* = 16–17 fish per treatment group; thin vertical lines in (a) and (b) are to help visual clarity only

State 3 and state 4 mitochondrial respiration rates increased with test temperature (state 3 *η* ^2^ = 0.049; state 4 *η* ^2^ = 0.036; Table 1; Figure 3a,b), but were not significantly modified by acclimation temperature or diet (Table 1). The interaction between acclimation temperature and diet modified P:O ratios (*η* ^2^ = 0.05; Figure 3c,d; Table 1). A higher acclimation temperature and infrequent feeding led to higher P:O ratios, but there was a larger increase in P:O ratio in infrequently fed and warm‐acclimated fish (Figure 3c,d). Test temperature had no effect on P:O ratio (Table 1).

### 3.3 Changes in aerobic scope did not reflect changes in scope for ATP production

Aerobic scope increased with increasing test temperature (main effect of test temperature; *η* ^2^ = 0.26), but it was not modified significantly by acclimation temperature or diet (Table 1; Figure 2c,d). Similarly, ATP production scope (i.e., aerobic scope × P:O ratio; ATP_scope_; Figure S1) increased with test temperature (*η* ^2^ = 0.19; Table 1, Figure 3e,f). However, ATP production scope was also greater in infrequently fed and in warm‐acclimated fish, with a significant acclimation*diet interaction (*η* ^2^ = 0.04) indicating that the capacity to increase ATP production was increased to a greater extent in warm‐acclimated infrequently fed fish (Table 1).

### 3.4 Latency to leave the refuge area did not change with feeding or temperature changes

Latency to leave the refuge was variable but not affected significantly by any of the experimental factors (Figure S2a,b; Table 2).

**TABLE 2:** Results from the analysis of behavior. Results from the permutational analysis are shown for latency to leave refuge (latency), time spent on exploration (time), and total distance explored (distance). Numerator degrees of freedom = 1 for all factors and interactions (Test = acute test temperature; Acc = acclimation temperature; Diet = frequency of feeding), and sample sizes were 16–17 fish per treatment group. Hypothesized relationships between metabolic traits and behavior (see Introduction) were analyzed by analysis of covariance with resting oxygen consumption (MO_2rest_), resting ATP production (ATP_rest_), aerobic scope (MO_2scope_), and scope for ATP production (maximum‐resting; ATP_scope_) as covariates. We predicted that the relationships should be more pronounced in infrequently fed fish, and we therefore analyzed frequently (Freq) and infrequently (Infreq) fish separately. Significant results are shown in bold; see main text for more details of analysis

Source	Latency		Time		Distance	
TestT	0.75		**0.0018**		**<0.001**	
AccT	0.94		**0.0014**		**0.0040**	
Diet	0.12		0.94		0.30	
TestT:AccT	0.071		0.55		**<0.001**	
TestT:Diet	0.29		0.47		0.64	
AccT:Diet	1		0.98		**0.016**	
TestT:AccT:Diet	0.42		0.35		0.090	

Covariates	Freq	Infreq	Freq	Infreq	Freq	Infreq
MO_2rest_	0.21	0.17	0.18	**0.0036**	0.27	**0.0058**
ATP_rest_	0.90	0.17	0.98	**0.012**	0.82	**0.019**
MO_2scope_			0.98	0.097	0.15	0.11
ATP_scope_			0.10	**0.013**	0.26	**0.019**

### 3.5 Exploration depended of feeding frequency and temperature

**FIGURE 4:**
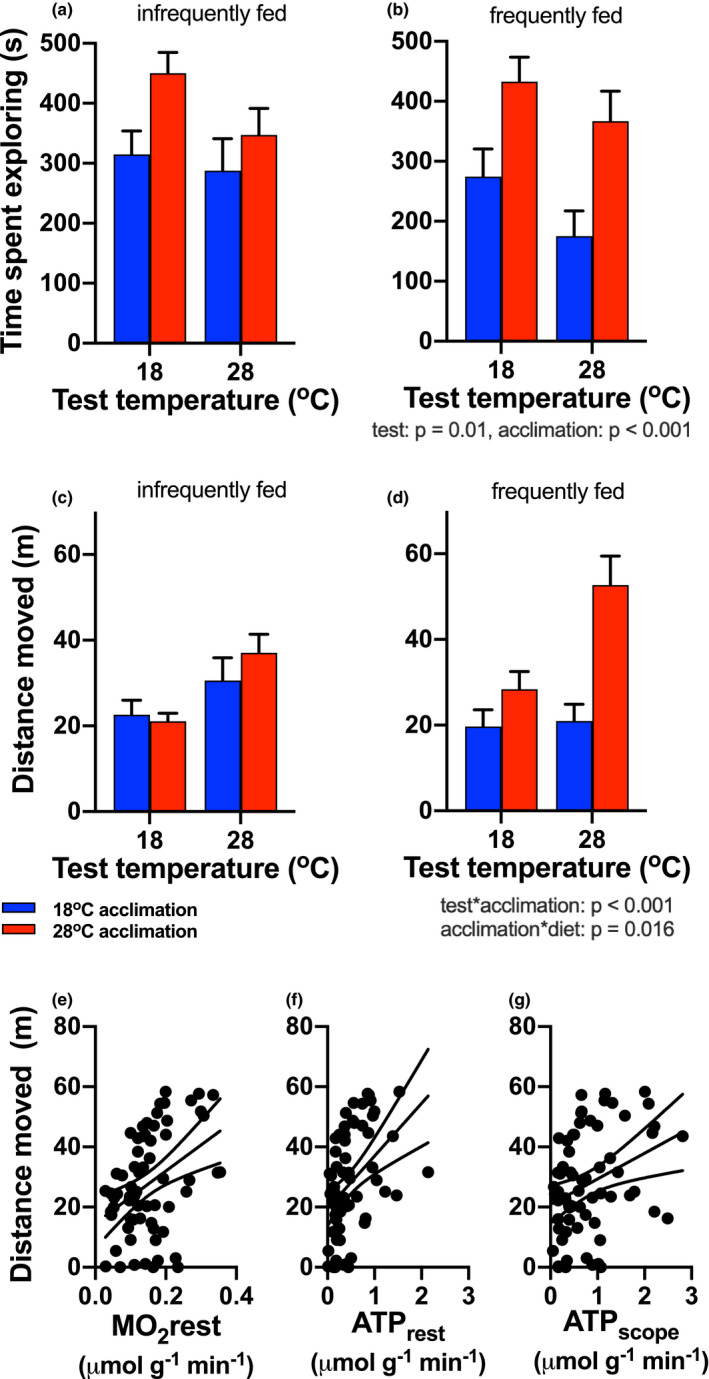
Exploration behavior. The time spent exploring in the arena (a, b) decreased with increasing test temperature (test temperature main effect), and warm‐acclimated fish (red bars) spent more time exploring than cold‐acclimated fish (blue bars; main effect of acclimation). The total distance traveled by fish in the arena (c, d) was determined by significant interactions between test and acclimation temperature, and between acclimation temperature and diet. Statistical results are summarized below the right panels of each behavioral response. Distance moved increased linearly with resting oxygen consumption (MO_2rest_) (e), resting ATP production (ATP_rest_) (f), and ATP production scope (ATP_scope_) (g). Significant regression lines (±95% CI) are shown (MO_2rest_: Y = 14.66 + 86.55x, *R* ^2^ = 0.16; ATP_rest_: Y = 19.47 + 17.48x; *R* ^2^ = 0.19; ATP_scope_: 20.80 + 8.55x; *R* ^2^ = 0.11). Means ± *SE* are shown in (a–d), and sample size was *n* = 16–17 fish per treatment group. Data from individual infrequently fed fish across all factors are shown in (e–g)

Time spent exploring (Figure 4a,b) by fish increased with increasing test temperature (main effect, *η* ^2^ = 0.037; Table 2), and it was greater in warm‐acclimated fish (main effect of acclimation, *η* ^2^ = 0.12; Table 2). Time spent exploring was not affected by diet (Table 2).

The total distance moved by fish in the exploration arena was greatest in warm‐acclimated and frequently fed fish (Figure 4c,d; Table 2). Warm‐acclimated, frequently fed fish at 28°C test temperature moved by far the greatest distance, which explains the interactions between test and acclimation temperatures (*η* ^2^ = 0.037), and acclimation temperature and diet (*η* ^2^ = 0.045; Table 2).

### 3.6 Metabolic traits predict behavior only in infrequently fed fish

Metabolic traits covaried with behavior only in infrequently fed fish (Table 2; Figure 4e,f). Resting oxygen consumption rate, resting ATP production rate, and scope for ATP production covaried with time spent exploring the arena and total distance moved (Table 2; Figure 4e,f). However, latency to leave the refuge did not covary with any metabolic trait, and aerobic scope was not correlated with any behavioral trait (Table 2; Fig. S3).

## 4 DISCUSSION

We accept our hypothesis predicting that infrequent feeding increases mitochondrial efficiency, particularly in warm‐acclimated fish. However, infrequent feeding did not necessarily lead to increased boldness and exploration as predicted. Nonetheless, in infrequently fed individuals resting oxygen consumption rates and ATP production scope were positively correlated with exploration as predicted. However, contrary to our prediction, resting ATP production was positively related to distance moved in the exploration arena. We accept the hypothesis that cold‐acclimated animals had lower mitochondrial efficiency, with the difference being especially marked in infrequently fed fish, and that aerobic scope did not reflect this change in ATP production. Aerobic scope also was not correlated with any of the behavioral traits.

Fasting is known to increase mitochondrial efficiency, thereby maximizing ATP production (Monternier et al., 2014; Salin et al., 2018). Our fish showed similar responses even though they were not fasting, indicating that a more moderate reduction in food availability leading to a negative energy balance is sufficient to elicit compensatory changes in mitochondrial efficiency. Both infrequent feeding and higher temperatures experienced by warm‐acclimated fish can lead to decreases in animal energy status resulting from reduced energy intake and increased expenditure, respectively. In our study, either treatment increased mitochondrial efficiency, but their combined effect caused the greatest increase. There may also be an increase in oxidative stress associated with increasing P:O ratio (Korshunov, Skulachev & Starkov 1997; Roussel & Voituron, 2020), so that there is a trade‐off between more efficient ATP production and cellular damage. For example, increased capacity for ATP production in fasting brown trout (*Salmo trutta*) was paralleled by an increase in reactive oxygen species production (ROS) (Salin et al., 2018). Additionally, cold acclimation can lead to increased ROS production and lipid peroxidation in mosquitofish (*Gambusia holbrooki*) (Loughland & Seebacher, 2020). An increase in ROS resulting from the combined effects of infrequent feeding and cold acclimation may prevent increases in mitochondrial efficiency, although this suggestion must be tested experimentally.

The increase in mitochondrial efficiency in warm‐acclimated, infrequently fed fish was not accompanied by any change in substrate oxidation rate or proton leakage rate, indicating that relative changes in these rates are not responsible for the observed increase in efficiency. Increased P:O ratio could result from an increased number of protons pumped across the inner membrane for each pair of electrons transferred (H^+^/2e^−^ ratio) (Brand, 2005). Such an increase in H^+^/2e^−^ ratio can result from a decrease in the slip reactions that occur in complexes of the respiration chain, particularly complex IV (Kadenbach, 2003). Another mechanism that could lead to increased P:O ratio is an increase in ATP synthase efficiency, which manifests as a decrease in the amount of H^+^ needed to flow through complex V to synthesize a molecule of ATP (Brand, 2005).

Our results show that aerobic scope does not change statistically across diet and acclimation treatments. The typical interpretation of such results would be that the energy available for activity does not vary with food availability or acclimation temperature. However, in warm‐acclimated fish, low food availability almost doubled mitochondrial efficiency compared to high food availability (and compared to cold‐acclimated fish). Calibrating individual oxygen consumption rates by their mitochondrial efficiency reveals that warm‐acclimated, infrequently fed fish produce far more ATP than predicted by aerobic scope.

Aerobic scope has been used widely as a proxy for the amount of energy available for activities beyond cellular maintenance (Norin & Metcalfe, 2019). Individuals with greater aerobic scope are considered to have greater performance and fitness under given environmental conditions. Many ecological and evolutionary theories about animal energetics have been based on whole‐animal oxygen consumption measures (Brown et al., 2004). Variation in behavioral traits too has often been explained by differences in oxygen consumption rates (Metcalfe et al., 2016), and aerobic scope is considered to be an important enabling mechanisms underlying behavior. In particular, boldness and exploration (Le Galliard et al., 2012) are often positively associated with aerobic scope, and bolder individuals had higher aerobic scope in flatfish (Rupia et al., 2016) and bluegill sunfish (Binder et al., 2016). The concept of the fast‐slow life‐history continuum (Gaillard et al., 1989) and the pace‐of‐life syndrome hypothesis at the intra‐species level (Reale et al., 2010) suggest that individuals with high metabolic (oxygen consumption) rates should exhibit a series of matching life‐history and behavioral traits (Reale et al., 2010). In particular, individuals with higher metabolic rates are predicted to be bolder and more exploratory, supposedly because a large “metabolic machinery” is necessary to provide energy for a proactive lifestyle (Careau et al., 2008). Zebrafish can have consistent proactive (“bold”) and reactive (“shy”) personality types, and proactive personality types can be associated with higher resting metabolic rates (Yuan et al., 2018). Nevertheless, high oxygen consumption does not always correlate with boldness or exploration, and the strength of the relationship between oxygen consumption and behavior seems to vary across environmental gradients (Killen et al., 2011; Metcalfe et al., 2016). It is mechanistically more consistent that energetically expensive behavioral phenotypes correlate with efficient ATP production rather than high oxygen consumption rates. The sensitivity of mitochondrial efficiency to environmental variation can explain why correlations between metabolic traits and behavior are dependent on environmental conditions.

High rates of energy use (oxygen consumption) at rest as a result of relatively inefficient mitochondria would require greater need to forage and may therefore be correlated with boldness and exploration (Krause et al., 1998), particularly when food is scarce (Killen et al., 2011). Our data support this suggestion in infrequently fed fish. On the other hand, high energy use during activity (i.e., high aerobic scope) would increase cost of transport and therefore be more likely to correlate negatively with exploration and activity (Jahn & Seebacher, 2019). Conversely, more efficient ATP production by mitochondria would support movement and lead to a positive association with exploration and possibly dispersal. Even though our data support these hypotheses at least partially, except that aerobic scope was not correlated with behavioral traits, there are some points to note. The pace‐of‐life syndrome and the fast‐slow life‐history continuum, as well as our correlations are just that, correlations. To resolve cause‐and‐effect relationships, the field must progress to manipulative experiments (Killen et al., 2017). The results from our factorial experiment are more informative than the correlations in revealing the environmental and physiological contexts that influence behavior. For example, high temperatures and food availability promoted exploration distance. It may be tempting to conclude that low energy status (low temperature and infrequent feeding) constrains movement. However, ATP production is as high or higher in infrequently fed compared to frequently fed fish so that the difference in behavior is not simply related to energetics. Behavioral and metabolic traits may each be linked to other traits such as endocrine signaling or muscle function, so that the correlation between behavior and metabolism does not reflect a mechanistic link. Our correlations, even though significant, explained only a small proportion of variation in behavioral traits, which confirms that it would be rewarding to also focus on other traits than metabolism to explain variation in behavior. It is interesting to note that “time to leave refuge” (boldness) was not correlated with oxygen consumption rates nor ATP production rates, which is at variance with the notion that individuals with higher resting oxygen consumption rate are bolder (Biro & Stamps, 2010). Boldness has also been linked to levels of glucocorticoids (Raoult et al., 2011), and glucocorticoid signaling also modulates metabolism (Sacta et al., 2016). It is possible therefore that the correlation between behavior and metabolism does not reflect a casual relationship, but instead results from the dependence of each on glucocorticoids. Correlations between behavior and other traits should therefore be confirmed by manipulative experiments.

Our data emphasize the importance of mitochondrial bioenergetics in interpreting ecological and behavioral relationships. There is an important distinction between energy use and ATP production. The former reflects the requirements for environmental resources, but does not necessarily represent the energy available for activity. Ecologically important behaviors such as exploration and possibly dispersal therefore rely both on resource availability and efficiency in energy transduction. Combining ecological and physiological approaches in parallel will be more effective to increase understanding the impacts of environmental changes on animal movement.

## CONFLICT OF INTEREST

The authors declare no competing interests.

## AUTHOR CONTRIBUTIONS

**Amélie Le Roy:** Data curation (equal); Formal analysis (equal); Investigation (equal); Writing‐review & editing (equal). **Geoffrey P. F. Mazué:** Investigation (equal); Methodology (equal); Writing‐review & editing (equal). **Neil B. Metcalfe:** Conceptualization (equal); Writing‐review & editing (equal). **Frank Seebacher:** Conceptualization (equal); Formal analysis (equal); Funding acquisition (equal); Methodology (equal); Project administration (equal); Resources (equal); Supervision (equal); Writing‐original draft (equal); Writing‐review & editing (equal).

## Supporting information

Supplementary Material

## Data Availability

Data are available in Dryad (https://doi.org/10.5061/dryad.rxwdbrv8k).
