# Supplementary material for: Diet and temperature modify the relationship between energy use and ATP production to influence behavior in zebrafish (Danio rerio)
Source: Ecol Evol. 2021 Jun 21;11(14):9791–803. doi: 10.1002/ece3.7806 (PMC8293724; doi:10.1002/ece3.7806)
Supplement: Supplementary file 1 — Supplementary Material [file ECE3-11-9791-s001.docx]

Supporting Information

**Table S1** Indices of fish condition. Values for a refinement of Fulton’s K condition factor (g cm^-2.55^) are shown before and after each of the four 3-week diet and thermal acclimation treatments (AccT, acclimation temperature). Data are shown as mean ± s.e.m. Sample size was n = 16-17 fish per treatment.

|  | Infrequently fed fish | | Frequently fed fish | |
| --- | --- | --- | --- | --- |
|  | Acc 18°C | Acc 28°C | Acc 18°C | Acc 28°C |
| Before treatment | 2.34 ± 0.08 | 2.26 ± 0.07 | 2.33 ± 0.08 | 2.29 ± 0.06 |
| After treatment | 2.06 ± 0.07 | 2.01 ± 0.09 | 2.77 ± 0.08 | 2.57 ± 0.10 |

**Figure S1** Estimated ATP production at rest (ATP_rest_) and during activity (ATP_max_) plotted against oxygen consumption. Each data point represents one fish, and data from all treatments are shown together.

**Figure S2** Behaviour of fish. None of the experimental factors had a significant effect on latency to leave the refuge [(a) infrequently fed; (b) frequently fed]. Means ± s.e. are shown and sample size was n = 16-17 fish per treatment group.

**Figure S3** Significant covariation between metabolic traits and behaviour in infrequently fed fish. Resting oxygen consumption (MO_2rest_) (a), resting ATP production (ATP_rest_) (b), and ATP production scope (ATP_scope_)(c) were significant covariates in the factorial analysis of time spent exploring by infrequently fed fish in the arena. However, there was no significant linear relationship across all factors between these metabolic parameters and time spent exploring. Data from individual infrequently fed fish across all factors are shown.
